# Reliability and validity of the Norwegian child and parent versions of the DISABKIDS Chronic Generic Module (DCGM-37) and Diabetes-Specific Module (DSM-10)

**DOI:** 10.1186/1477-7525-10-19

**Published:** 2012-02-02

**Authors:** Dag Helge Frøisland, Trond Markestad, Tore Wentzel-Larsen, Torild Skrivarhaug, Knut Dahl-Jørgensen, Marit Graue

**Affiliations:** 1 Research Center for Child and Youth Competence Development, Lillehammer University College, Lillehammer, Norway; 2 The Norwegian Childhood Diabetes Registry,Oslo University Hospital, Ullevål, Oslo, Norway; 3 Department of Clinical Medicine, University of Bergen, Bergen, Norway; 4 Department of Research, Innlandet Hospital Trust, Brumunddal, Norway; 5 Centre for Child and Adolescent Mental Health, Eastern and Southern Norway, Oslo, Norway; 6 Norwegian Centre for Violence and Traumatic Stress Studies, Oslo, Norway; 7 Department of Paediatrics, Oslo University Hospital Ullevål, Oslo, Norway; 8 Oslo Diabetes Research Centre, Oslo University Hospital, Oslo, Norway; 9 Faculty of Medicine, University of Oslo, Oslo, Norway; 10 Centre of Evidence- Based Practice, Bergen University College, Bergen, Norway; 11 Department of Paediatrics, Haukeland University Hospital, Bergen, Norway; 12 Pediatric Department, Innlandet Hospital Trust, Lillehammer, Norway

**Keywords:** Health-related quality of life, Type 1 diabetes, Children, Adolescents, Psychometrics, Reliability, Validity, DISABKIDS

## Abstract

**Background:**

International guidelines on type 1 diabetes advocate routine screening of health-related quality of life (HRQOL). DISABKIDS questionnaires are the first instruments developed across cultures and nations to provide age-appropriate measures of HRQOL in children with chronic diseases. DISABKIDS includes a Chronic Generic Module 37 (DCGM-37) and disease-specific modules. The purpose of this study was to examine reliability and validity of the Norwegian versions of the DISABKIDS questionnaires in children and adolescents with type 1 diabetes.

**Methods:**

The DCGM-37 and the Diabetes Specific Module-10 (DDM-10) were translated into Norwegian using standard forward-backward translation. Eight to 19 year old children and adolescents with type 1 diabetes scheduled for routine follow-up at three diabetic clinics in Norway and one of their parents were invited to complete the DCGM-37 and the DDM-10. Internal consistency was determined using Cronbach's alpha. Results were compared with those of the Child Health Questionnaire Children Form-87 (CHQ-CF87) and Child Health Questionnaire Parent Form-50 which are established generic questionnaires. DISABKIDS results were related to age, gender, duration of diabetes, mode of insulin delivery and metabolic control. Clinical data were obtained from the Norwegian Childhood Diabetes Registry.

**Results:**

Of 198 eligible child-parent dyads, 103 (52%) completed the questionnaires. Mean age was 13.6 (2.6), range 8-19 yrs, 52% were boys. Cronbach's alpha was > 0.70 for all the DISABKIDS sub-scales except two (physical ability and social inclusion). There were moderate to high correlations (0.65-0.81) between the DISABKIDS scales and mental/emotional sub-scales of CHQ-CF87. Increasing age and higher HbA1c were significantly associated with reduced HRQOL scores. Parents tended to score their child's HRQOL lower than the children/adolescents themselves.

**Conclusions:**

The study shows that the DISABKIDS instruments are applicable to a Norwegian childhood diabetes population. They seem to be a relevant supplement to other clinical indicators in medical practice and research.

## Introduction

Type 1 diabetes is one of the most common chronic diseases of childhood, and the incidence in Norway of 30 new cases per 100 000 person years is one of the highest in the world[1]. Diabetes poses significant every-day challenges since optimal blood glucose control is important in order to avoid severe acute complications (i.e. hypoglycaemia and diabetes ketoacidosis) and long term consequences, such as early onset of cardiovascular disease, visual impairments, renal failure, neuropathy and premature death[2,3]. The burden of diabetes on the children and their families is well known to affect both psychological and total wellbeing [4-6], and young persons with diabetes report impaired self-perceived health-related quality of life (HRQOL) [7,8]. A good quality of life is an important treatment goal in itself [9], but is also important in order to achieve other treatment goals [10-13]. The International Society for Pediatric and Adolescent Diabetes-(ISPAD) guidelines therefore advocate assessment of quality of life as important as screening for other complications related to diabetes [9,14]. HRQOL is a multidimensional construct including at least physical, psychological and social domains. To make international comparisons possible it is advocated that test instruments are developed in cross-cultural and cross-national study groups [15,16]. In line with this goal "The DISABKIDS project", which was funded by the European Commission, was developed in seven European countries with the purpose of developing instruments for assessing HRQOL of children with different chronic health conditions [16,17]. The DISABKIDS instruments consist of questionnaires which include a generic module (DISABKIDS Chronic Generic Module (DCGM-37)) and disease-specific modules (e.g. DISABKIDS Diabetes- Specific Module (DDM-10)) [18]. The DCGM-37 is the only HRQOL instrument developed across cultures for children with chronic diseases [19]. Due to its novelty, relatively few studies using DISABKIDS have been published so far, apart from the psychometric properties reported from the European field study [17]. A literature search disclosed no recent validation studies, but Swedish and Greek groups have published results on DISABKIDS data [19-21].

The aims of the present study were to examine reliability and validity of the Norwegian versions of the DCGM-37 and DDM-10 questionnaires when assessing HRQOL among children and adolescents with type 1 diabetes based on their own report and that of their parents. Internal consistency was assessed by Cronbach's alpha coefficient. Convergent validity was assessed by comparison with established generic instruments, in this case the Child Health Questionnaire Children Form-87 (CHQ-CF87) and Child Health Questionnaire Parent Form-50 (CHQ-PF50). We also evaluated the instruments' ability to discriminate between patients with different characteristics, i.e. age, gender, duration of disease, treatment modalities and metabolic control reflected in HbA1c. Finally, we studied whether the children and their parents assessed the child's HRQOL differently.

## Methods

### Participants

Except for families who were not able to speak or read Norwegian, all 8-19 year old children or adolescents with type 1 diabetes scheduled for follow-up at three pediatric departments in eastern Norway between October 1^st^, 2009 through February 28^th ^2010 and one of their parents were invited by mail to participate in the study before a scheduled consultation.

Whether the cohort in the present study was representative for Norwegian children and adolescents with diabetes was assessed by comparing demographic and clinical characteristics with that of the Norwegian Childhood Diabetes Registry, which is a population based, nationwide registry covering all pediatric departments in Norway. In 2010, 95% of all children and adolescents with diabetes treated by pediatricians were included in the registry [22].

### Instruments

#### DISABKIDS

The DISABKIDS Chronic Generic Module (DCGM-37) is a questionnaire which measures general HRQOL and the level of distress caused by a chronic disease, and can be supplemented with condition-specific modules for asthma, arthritis, cerebral palsy, cystic fibrosis, dermatitis, epilepsy and diabetes [18]. The instruments include one form to be filled in by children between 8 and 18 years of age, and one form for their parents. A four-week recall period is used for all items except item 11 "About symptoms" which has a one year recall in the diabetes specific module.

**Figure 1:**
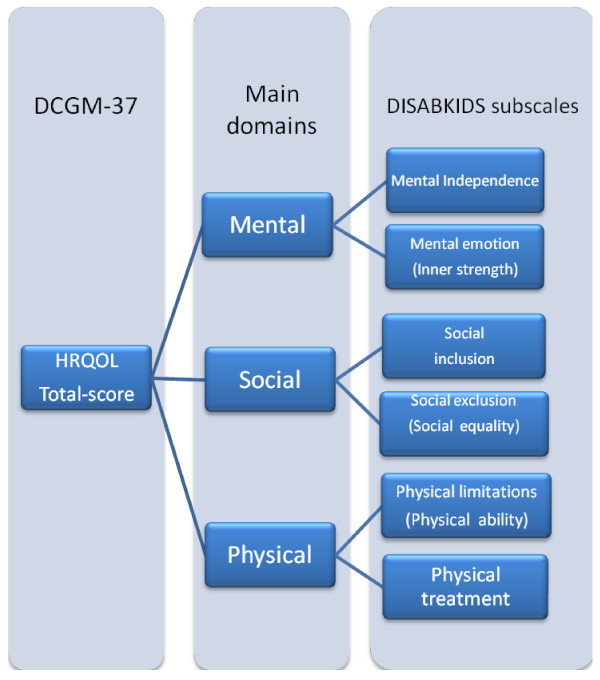
**The structure of the DISABKIDS Chronic Generic Module-37, (DCGM-37), included rephrased, positive subscales, (in parenthesis)**.

The DCGM-37 questionnaire contains 37 items which explore six dimensions of HRQOL [16,17] (Figure 1): *"*Mental independence" assesses whether the child feels confident about the future and is able to live an autonomous life without impairments caused by the condition, "Mental emotion" addresses emotional reactions, such as worries, concerns, anger and problems caused by the child's condition, "Social exclusion" deals with the feeling of being left out and stigmatized, "Social inclusion" focuses on positive social relationships and the understanding of others, "Physical limitation" refers to somatic limitations, due to the condition and "Physical treatment" assesses the impact of taking medication, receiving injections, etc.

Each item is scored on a five-point Likert scale indicating frequency of behaviours or feelings as 1 = never, 2 = seldom, 3 = quite often, 4 = very often, 5 = always. The scale for negatively worded items was reversed according to the manual. In computation of sum scores, missing values were substituted with the mean of non-missing items if only one item of the domain was missing. If more than one item was missing the domain was not scored. The sum score of each domain is the sum of the single item scores. From the raw score a total score may be computed with a range from 0 to 100 with higher scores indicating higher self-perceived HRQOL.

**Figure 2:**
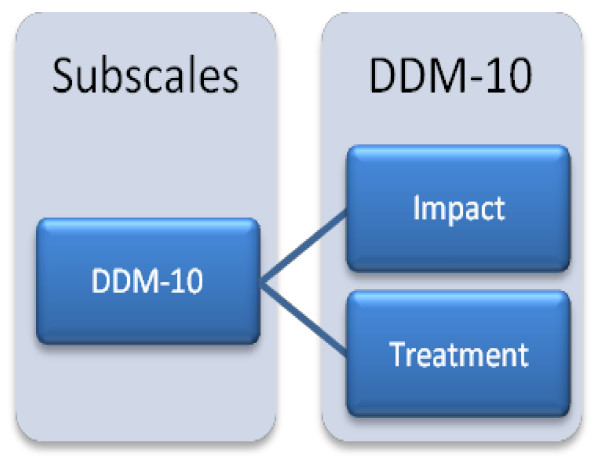
**The structure of the DISABKIDS Diabetes Module-10 (DDM-10)**.

The diabetes specific instrument (DDM-10) consists of an "Impact" and a "Treatment" scale (Figure 2). The "Impact scale" deals with emotional reactions of blood glucose control and adhering to diets in everyday life, and the "Treatment scale" deals with emotional reactions to the planning of treatment and the burden of carrying equipment. DDM -10 items are scored on a five-point Likert scale, and a 0-100 score is calculated for each sub-scale [23].

The DCGM-37 and DDM-10 forms were forward and backward translated from English to Norwegian according to an international scientific translations procedure[24].

The goal of this process was to keep the original meaning of the questions and simultaneously to find the most appropriate terms in the new language. The final versions were approved by the DISABKIDS research group.

A standard manual detailing the data collection was distributed to each of the three participating centres.

In the past, presentations of HRQOL results have been criticised for being incomprehensible in relation to clinical relevance [25]. To address this critique, Osobo et al have suggested that HRQOL results will be more meaningful if negative domains were reconceptualised to positive statements [20,26]. Therefore, in the following, similar to the presentation of results from Chaplin and colleges [20]"Mental emotion" is rephrased as "Inner strength", "Social exclusion" as "Social equality", and "Physical limitations" as "Physical ability".

#### Child Health Questionnaire

In additions to the DISABKIDS questionnaires, the children and adolescents were asked to complete the Child Health Questionnaire Form-87 (CHQ-CF87), and their parent the Child Health Questionnaire Parent form-50 (CHQ-PF50). The CHQ-CF87 is a generic HRQOL questionnaire designed to measure physical, emotional, behavioural and social well being [27]. From 10 years of age children were asked to complete the CHQ-CF87 independently, while the questions could be read to younger children[28,29].

Health is assessed over several domains i.e. general health perceptions, physical functioning, role/social- physical functioning, bodily pain, role/social- emotional and behavioural functioning, parent impact-time and parent impact-emotional, self-esteem, mental health, behaviour, family activities and family cohesion. The responses are indicated on 4 to 6 point Likert scales specifying level of agreement to a certain categorical statement such as "very often" to "not at all". The responses within each subscale are summed, and the raw scores are transformed to a score between 0 and 100, with higher scores indicating better functional health and well-being. Extensive studies on the psychometric properties of the CHQ-CF87 and CHQ-PF50 suggest strong internal consistency, content validity and construct validity. Translation to Norwegian has been carried out previously, and the instruments have been used in several Norwegian patient cohorts [30-32]. A four- week recall period is used for all scales except for the "Change in Health" and "Family Cohesion" items which refer to last year, and the "General health" scale which has no recall period.

The questionnaires were completed at the clinic when the participants met for their follow up. As recommended for CHQ-87, health care personnel were available to clarify questions for the age group 8-9 years, if necessary. The child/adolescent and their parent completed the questionnaire independently of each other.

The completed questionnaires were scanned using Tele Form (Cardiff software, Vista, CA) and checked for scanning errors.

#### Clinical characteristics

HbA1c was analyzed at the same visit as the questionnaires were filled in using Bayer DCA 2000 (Tarrytown, NY - normal reference range 3.4-6.1%). The incidence of reported ketoacidosis and hypoglycaemia was too low to allow analyses of HRQOL scores in relation to these clinical markers.

### Ethical considerations

The children and adolescents and their parents gave written consent according to Norwegian requirements. The study was approved by the Regional committee on medical research ethics.

### Statistical analyses

Results are presented as means with one standard deviation (SD) or as rates (percentages). Floor and ceiling effects are reported in numbers of patients with HRQL scores of 0 (floor) and 100 (ceiling). A percentage above 25 was characterized as high.

Internal consistency refers to the degree to which the different items in a scale measure the same construct. For the DISABKIDS questionnaires reliability was assessed by tests of internal consistency of each of the subscales and the overall sum score. Cronbach's alpha coefficients above 0.70 are generally viewed as acceptable when instruments are used for group level analyses [33,34]. With short scales as in DCGM-37 and DDM-10 it is often more appropriate to report mean inter item correlations. Upper and lower limits of mean inter item correlations are a matter of discussion. Some authors claim that values between 0.2 and 0.4 are optimal [35], while others argue that a mean inter item correlation consistently above 0.70, may indicate redundancy [36]. In the present article, we consider mean inter item correlations between 0.2 and 0.7 as satisfactory.

Convergent and divergent validity of the DISABKIDS questionnaires DCGM-37 and DDM-10 were assessed with reference to the generic questionnaires CHQ- CF87 and CHQ-PF50, respectively, using Pearson correlation adjusted for age and gender. A correlation coefficient (r) above 0.5 between measures of construct related to each other was considered as high and a coefficient between 0.3 and 0.5 as moderate convergence, while measures were not considered to be related if correlation coefficients were below 0.3 [34].

DISABKIDS' discriminant validity in relation to age, gender, duration of diabetes, mode of insulin delivery and metabolic control (i.e. levels of HbA1c) was assessed using multiple regression analysis.

Paired sample t-tests were used to assess associations between scores obtained by the children and their parents.

Significance was defined as p < 0.05. SPSS version 18.0 (SPSS IBM, NY, USA) was used for analyses.

## Results

Of 198 eligible child-parent dyads 103 (52%) completed the questionnaires. Mean age was 13.6 (2.6), range 8-19 years, and 53 (52%) were boys. Compared with the national diabetes cohort, the participants had similar gender distribution, mean age, mean duration of diabetes and mean body mass index (BMI), but somewhat lower mean HbA1c, lower average numbers of consultations and higher rate of insulin pump use (Table 1).

**Table 1:** Demographic and clinical characteristics of the children and adolescents included in the Norwegian Childhood Diabetes Registry and the study population.

	Norwegian Childhood Diabetes Registry (n = 2109)			Study cohort (n = 103)			p
	**Number** **examined**			**Number examined**			

HbA1c (%) - mean (SD)	2048	8.65	(1.4)	101	8.04	(1.1)	< 0.001

Boys - n (%)	2109	1114	(53)	102	53	(52.0)	0.92

Age(yrs)-mean (SD)	2109	12. 9	(3.8)	102	13.6	(2.6)	0.07

BMI kg/m^2^- mean (SD)	2081	20.4	(3.9)	100	20.8	(3.6)	0.29

Consultations last year - mean (SD)	2068	3.7	(1.7)	102	3.3	(1.3)	0.02

Diabetes duration (yrs) -mean (SD)	2109	5.2	(3.6)	102	4.6	(3.5)	0.06

Insulin pump - n (%)	2109	1073	(51)	102	74	(73)	< 0.001

Mean scores on the children's and adolescents' self report forms varied between 62 and 83. Very few had floor or ceiling values (Table 2).

**Table 2:** Subscale and total sum scores on the Norwegian self report version of DISABKIDS.

	**Scale**	**n**	**Mean score** **(1-100)**	**Standard** **Deviation**	**Floor/ceiling** **n/n**	**"Chronbach alpha"**	**Mean inter-item** **correlation**
	
*Mental*	Mental independence	103	78	13.7	0/7	0.75	0.33
	Mental Emotion (Inner strength)	103	77	15.6	0/8	0.85	0.47
	
*Social*	Social Inclusion	102	80	11.5	0/3	0.60	0.22
	Social exclusion (Social equality)	103	83	13	0/8	0.70	0.30
	
*Physical*	Physical limitations (Physical ability)	103	77	11.4	0/5	0.55	0.19
	Medication/Treatment	101	75	17.2	0/9	0.80	0.40
	
*Total score*	HRQOL	100	78	16.9	0/0	0.92	0.25
	
*Diabetes module*	Impact	102	70	16.9	0/6	0.79	0.41
	Treatment	100	62	20.7	1/5	0.79	0.49

All subscales are scored from 0 to 100 with higher scores indicating higher self-perceived HRQOL.

### Reliability

For the children's questionnaires the internal consistency of the DCGM-37, calculated as Cronbach's alpha, varied between 0.55 and 0.92 (Table 2.). Cronbach's alpha was above 0.70 for all subscales except for "Physical ability" and "Social inclusion". Mean inter item correlations were above 0.20 and below 0.50 for all subscales except for the "Physical ability" subscale which was 0.19. For the DDM-10 Cronbach's alpha was 0.79 for both scales, and mean inter-item correlations were 0.41-0.49.

For the parent's questionnaires, Cronbach's alpha varied between 0.74 and 0.89 for the DCGM-37 and was 0.83 for both scales on the DDM-10. Mean inter item correlations varied from 0.32 ("Physical ability") to 0.55 ("Inner strengths").

### Convergent validity

Correlation coefficients between the DCGM-37 and DDM-10 scales and the "Mental health" subscale in CHQ-CF87 were in the range 0.54-0.81 (Table 3). Correlation coefficients were in the range 0.65-0.81 between the DCGM-37 total score and six of the twelve subscales in the CHQ-CF87, and in the range 0.49-0.67 between "Role emotional" in CHQ-87 and the DCGM-37 "Treatment" scale and the DDM-10 ("Impact" and "Treatment" scales).

**Table 3:** Correlations^a ^between the DISABKIDS and the CFQ-CF87 scales in a cohort of 103 children and adolescents with type 1 diabetes

CHQ-CF87	DISABKIDS								
	
	DCGM-37							DDM-10	
	
	Mental Independence (Inner strength)	Mental Emotion	Social Excl	Social Incl (Equality)	Physical limitations (abilities)	Treatment	DCGM-37 sumscore	DDM impact	DDM treatment
Physical function	0.21	0.15	0.21	0.21*	0.06	0.20	0.22 *	0.19	0.19

Role emotional	0.30**	0.39***	0.28**	0.27*	0.36**	0.59***	0.81***	0.67***	0.49***

Role behavioral	0.20	0.23*	0.15*	0.11	0.32**	0.23*	0.27*	0.22*	0.19

Role physical	0.26*	0.18	0.21*	0.17	0.34***	0.22*	0.29**	0.30**	0.15

Bodily pain	0.47***	0.51***	0.56***	0.49***	0.54***	0.49***	0.65***	0.38***	0.22*

Behavior	0.56***	0.59***	0.45***	0.45***	0.64***	0.60***	0.70***	0.54***	0.58***

Global behavior	0.26*	0.32**	0.23*	0.315**	0.32**	0.19	0.34***	0.26*	0.05

Mental health	0.69***	0.71***	0.58***	0.59***	0.71***	0.57***	0.81***	0.54***	0.55***

Self-esteem	0.60***	0.58***	0.52***	0.58***	0.64***	0.50***	0.72***	0.51***	0.44***

General health	0.54***	0.59***	0.54***	0.55***	0.60***	0.50***	0.70***	0.51***	0.44***

Change in Health	0.21*	0.25*	0.13	0.16	0.23*	0.07*	0.22*	0.11	0.11

Family activity	0.32*	0.33***	0.24*	0.14	0.22*	0.34***	0.35***	0.34***	0.38***

Family cohesion	0.33***	0.26*	0.35***	0.36***	0.45***	0.31**	0.43***	0.26*	0.28**

^a ^Pearsons's correlation coefficient adjusted for age and gender

*p < 0.005. **p < 0.01. ***p < 0.001

Correlation coefficients between the DCGM-37 scales and the CHQ-CF87 scales "Physical function" "Role behavioral", "Global behavior" and family related dimensions were low.

### Discriminant validity

The generic as well as the diabetes-specific module of DISABKIDS discriminated between age groups and levels of HbA1c (Table 4). Higher age and increasing HbA1c were associated with lower HRQOL scale scores.

**Table 4:** Effects of age and HbA1c on total and self reported scores in DISABKIDS.

	Age					HbA1c			
	**Subscale**	**Unadjusted effect**	**p**	**Adjusted** effect^a^*	**P**	**Unadjusted effect**	**p**	**Adjusted** effect^a^**	**p**

*Total sum- score*	HRQOL	*-0.94*	*0.08*	*-0.94*	*0.04*	*-2.49*	*0.01 *	*-2.40*	*0.01*

*Mental*	Mental independence	*-0.78*	*0.15*	*-0.87*	*0.13*	*-0.58*	*0.64*	*0.62*	*0.62*

	Mental Emotion (Inner strength)	*-1.58*	*0.01*	*-1.684*	*0.01*	*-2.77*	*0.05*	*-2.73*	*0.05*

*Social*	Social inclusion	*-0.75*	*0.11*	*-0.61*	*0.21*	*-2.49*	*0.02*	*-2.26*	*0.03*

	Social exclusion (Social equality)	*-0.57*	*0.29*	*-0.48*	*0.37*	*-2.16*	*0.06*	*-2.02*	*0.09*

*Physical*	Physical limitations (Physical Ability)	*-0.78*	*0.09*	*-0.91*	*0.05*	*-3.19*	*0.001*	*-3.39*	*0.001*

	Medication/Treatment	*-1.27*	*0.07*	*-0.97*	*0.19*	*-3.77*	*0.01*	*-3.37*	*0.03*

*Diabetes module*	Impact	*-1.47*	*0.03*	*-1.38*	*0.04*	*-4.43*	*0.002*	*-4.38*	*0.002*

	Treatment	*-3.01*	0.001*	*-2.84*	*0.001*	*-3.44*	*0.06*	*-2.54*	*0.16*

^a^Adjusted for gender, duration of diabetes and use of insulin pump vs. multi-injections using multiple linear regression analyses

*Effect per year of age, **Effect per % increment of HbA1c.

All subscales are scored from 0 to 100 with higher scores indicating higher self-perceived HRQOL.

Boys tended to score higher than girls, and children using insulin pump higher than those on multi-injections on total sum-scores and scores on most subscales. With respect to treatment modality, the differences in mean score between insulin pump and multi-injection users were between 3.5-4.0 on "Social equality "and "Physical treatment" subscales on DCGM-37 as well as for both subscales in the DDM-10 (results not shown).

There were no differences in scores with regard to duration of diabetes. Twelve (12%) of the participants had diabetes duration of less than one year. This group scored slightly higher on all subscales as well as on the HRQOL total score, but these differences were not statistically significant.

### Comparison of children's and parents' scores

Generally, children and adolescents tended to give higher scores than their parents. This was true for all subscales in DCGM-37 and for the "Impact" scale in DDM-10. Significant mean (SD) differences were found for the DCGM-37 total score (78 ± 11.0 vs. 76 ± 11.1, p = 0.03), the subscales "Inner strength" (77 ± 15.8 vs. 72 ± 13.1, p < 0.01) and "Social inclusion" (80 ± 11.7 vs. 74 ± 15,0 p < 0.001). In the DDM-10 "Treatment" scale parents tended to score higher than their children (66 ± 17.1 vs. 63 ± 20.4 p = 0.14).

## Discussion

Applied to this Norwegian child and adolescent population with diabetes and their parents the internal consistency reliability of the DISABKIDS instruments was satisfactory for all except two scales judged by Cronbach's alpha. Furthermore, the scores on the DCGM-37 subscales showed moderate to high correlations with the "Mental health" subscale, but low correlations with the "Physical function" and family related subscales on the previously validated CHQ-CF87 and CHQ-PF50 questionnaires. The DISABKIDS instruments discriminated between groups based on metabolic control (HbA1c) and age in that increasing HbA1c and age were associated with lower HRQOL, while no significant differences were found with respect to gender, duration of diabetes or insulin pump vs. multi-injections treatment, although there was a tendency that pump-users rated the impact of disease less and social equality higher than those on multi-injections. Parents generally scored their children's HRQOL lower than the children and adolescents themselves.

### Strengths and limitations

The major strength of the study was that the DISABKIDS questionnaires were applied to a wide age-range of children and adolescents (8-19 years old). Also, a broad perspective was taken by the collection of both self-reported and parent data. Furthermore, the results of the DISABKIDS were compared with those obtained with a HRQOL instrument (CHQ) which is well validated in Norwegian populations. Major weaknesses were the limited sample size and low participation rate. The reasons why 48% of the total population did not participate were multifactorial. The main reason reported by the nurses at the participative centers was that not all families were approached during periods with large work load in the clinic. However, this happened at random and we are confident that it did not introduce a bias.

Compared to the children registered in the national diabetes registry the study cohort had a somewhat lower mean HbA1c and higher proportion of insulin pumps users, but we still suggest that the results are representative of the national cohort for the following reasons: The difference in mean HbA1c was probably too small to be of major significance, and the difference in proportion of pump users was likely due to differences in treatment traditions. In Norway, pumps are, in practice, available without extra charge for all children, and the proportion choosing pumps is mainly a result of how familiar and confident the medical staff is with this modality. The clinics participating in the study have an active approach to encourage the use of pump, and the proportion of pump users in the study group was similar to the proportion among all the patients followed in the clinics.

Due to the relatively small sample size we did not perform factor analyses to assess the factor structure of the DCGM-37 or DDM-10 instruments, which is advocated when the instrument is applied to a larger population. With regard to reliability the study may be criticised for not applying test- retest reliability scores. However, this was not included because of concern for the patients and logistic challenges.

It is suggested that the CHQ-CF87 can be read to children less than ten years of age. The medical staff, after piloting, reported that some children between 8-9 years had insufficient reading skills to respond reliably on the DISABKIDS. Use of the instruments might therefore have a limitation in the youngest population if the questions are not read to them.

### Internal consistency

Most of the sub-scales showed Cronbach's alpha coefficients above 0.7, which are in agreement with the European field study [17]. The "Physical ability" scale had a low Cronbach's alpha compared to what was reported in the European field study where the patient populations consisted of seven different chronic conditions [17]. The "Physical ability" items in the DCGM-37 range from questions about difficulties with moving and running to questions on how life is ruled by the condition. Young persons with diabetes rarely experience physical complications due to the disease and, therefore, usually have no physical limitations. However, they experience significant practical and often emotional challenges due to repeated blood glucose measurements and administration of insulin, fear of hypoglycaemia, ketoacidosis and long term complications on a daily basis. Therefore, a low Cronbach's alpha was not unexpected. The Cronbach's alpha was also low for the "Social inclusion" subscale. In line with earlier reports, we suggest that the demands of adhering to treatment may create a feeling of separation from peers [20]. Also, differences in the experiences between users of pump and multi-injection treatments may create an incoherent scoring, explaining the lower alpha on this scale.

In general, few items in a scale, such as six items in the DISABKIDS subscales, may make Cronbach's alpha calculations vulnerable to variations between items, and mean inter-item (MII) correlation has been suggested as an alternative analysis of consistency [35]. This method modifies the findings in our study, as only the "Physical ability" subscale had a MII correlation below the lower acceptable limit of 0.2 underscoring that this subscale may not be informative for young people with diabetes. Furthermore, no scales in the two questionnaires had a MII above 0.50, strongly suggesting that the items in the scales were not redundant.

On the parents' reports Cronbach's alpha was above 0.7 on all subscales, and consistently higher than those of their children. Still, the pattern was the same as for their children in that the same subscales "Physical ability" and "Social inclusion" had the lowest Cronbach's alpha. These findings are in accordance with the European study [24].

The heading in both the child's and parent's form "About your typical day" may not be explicit enough for young people to connect it to "Physical ability". Parents, however, may have interpretive abilities that cause them to answer more consistently.

### Convergent validity

Considering the content of the DISABKIDS questionnaires, some scales were expected to correlate better than others with the CHQ-CF87 scales. The pattern of associations between the subscales of the two instruments largely supported the validity of the DISABKIDS instruments. The European validation procedure used only a few of the questions from CHQ-CF87 (personal communication, John Chaplin, 2009). As far as we know a comparison with complete CHQ questionnaires has not been done earlier when examining the validity of DISABKIDS.

Six of the subscales from CHQ-CF87 showed high correlation with DCGM -37 total score. This is as expected since these six subscales, "Role emotional", "Bodily pain", "Behavior", "Mental health", "Self esteem" and "General health" all have items that are similar to those in the DCGM-37 Questionnaire. On the other hand, the DCGM-37 does not have any sub-scales mirroring the "Change in health", "Family activity" or "Family cohesion". It is therefore appropriate that these scales in CHQ-CF87 showed low correlation with the subscale scores in DCGM-37. This feature may have clinical and scientific implication for children with diabetes. Other instruments than DISABKIDS may therefore be more applicable if the primary goal is to measure family related factors [37,38]. Furthermore, clinicians and researchers need to be aware that the DISABKIDS instruments seem to have their strength in measuring the mental and emotional aspects rather than detecting physical health and family related aspects of HRQOL.

The different subscales of the two instruments demonstrated correlations that may seem surprisingly high or low with reference to how they are named. However, when considering the content of the respective items in the DCGM-37 and CHQ-CF87, associations were mainly as expected. For example, the names of the subscales "Physical ability" in the DCGM-37 and "Physical function" in the CHQ-CF87 give the impression that they measure similar functions. However, the "Physical function" subscale in CHQ-CF87 measures limitation in nine specific physical activities due to health problems, while DCGM -37's "Physical ability" scale is constructed differently, e.g.-items like "Is your life ruled by you condition" and "Does it bother you that you have to explain to others what you can and can't do?" measure emotional reactions to living with impairments, while the CHQ-CF87 to a larger extent addresses physical limitations. The "Physical ability" subscale in DCGM-37 correlated best with "Mental health" in CHQ-CF87, suggesting that the six questions measure a wider construct than physical abilities alone.

"Inner strength" (DCGM-37) correlated well with "Mental health" (CHQ-CF87) as would be expected due to the construction of single items in the two scales. The same was true for "Mental independence" in DISABKIDS vs. "Mental health" and "Self esteem" subscales in CHQ-CF87. The three constructs "Mental health", "Self esteem" and "General health" in CHQ-CF87 have 40 items covering most of the 37 item DISABKIDS questionnaire. The DCGM-37 therefore seems to be well suited for measuring the mental and emotional burden of a disease like diabetes.

The two DDM-10 subscales "Impact" and "Treatment" and correlated well with the "Role emotional" subscale on the CHQ-CF87. The two DDM-10 subscales actually measure the emotional consequences of having a chronic illness, quite similar to what "Role emotional" measures. The "Treatment scale" in DCGM-37 and the "Role emotional" scale were similarly correlated. These findings suggest that the DISABKIDS instruments are suitable for an early detection of mental and emotional worries with possible negative influence on self-management. The fact that these instruments are available as computer programs and can be completed by the patient and automatically scored prior to consultation, make them particularly attractive in clinical practice [39].

### Discriminant features

The DISABKIDS generic instrument discriminated between patients based on age and metabolic control. It is likely that the scores on both of these variables have clinical significance as well [34]. Older children scored lower than the younger, a finding that is consistent with other studies on HRQOL using different instruments [31,40]. The effect of age may indicate a higher level of stress during puberty and late adolescence because of greater responsibility for own disease management and better cognitive ability to understand possible consequences of the disease, while parents, by taking more responsibility, tend to relieve the younger children from psychosocial burden [40].

The finding of higher HRQOL score with better metabolic control is also in accordance with earlier studies [41]. The fact that the DISABKIDS instruments are able to identify these differences is important, and of interest for both clinical work and further studies.

The reason why we did not find significant differences in scores related to gender, type of treatment or duration of diabetes may be due to lack of statistical power because of limited sample size. The trends were, however, similar to what has been found as statistical significant differences in larger studies [41].

When evaluating differences in HRQOL studies, it is equally important to evaluate the statistical findings and numeric differences in scores in relation to clinical importance. We believe that the statistically significant findings in our study are clinically significant as well. Generally, it has been suggested that on a scale of 0-100 a change of 5-10 points is clinically significant for an individual [34]. The numeric differences between those using insulin pump and multi-injections on the subscales "Impact" and "Treatment" on DDM-10 and on "Social equality" on the DCGM-37 may be clinically important, although they were not statistically significant in this limited study. The "Social equality" subscale consists of questions regarding external stigma. It has been reported that pump users have the ability to "hide" their disease better than those in need of other equipment for insulin delivery [42]. The feelings of less impact of disease and smaller problems related to treatment might therefore contribute to the pump users scoring higher on this subscale.

In agreement with earlier HRQOL studies we did not exclude diabetes patients with onset of disease less than one year prior to the study[40]. Patients with diabetes less than one year had non-significantly better HRQOL scores than those with longer duration. The lack of substantial difference may partly be due to the fact that most of those with duration less than one year had duration more than 6 months.

The DISABKIDS generic and diabetes specific modules showed differences between the children's and parent's score. The same findings, with parents tending to perceive their children's HRQOL lower than the children themselves, have been reported previously, for DISABKIDS as well as for other HRQOL instruments, and for other chronic diseases [20,43,44]. These findings may be due to changes in conceptualization of health related quality of life over the course of the disease trajectory towards better acceptance with duration of the disease [31,45]. It is also notable that the reverse was found on the DDM-10 "Impact scale", i.e. that disease was felt to have less impact by the parents than by their children or adolescents. The reason for this finding is not obvious, but may at least partly be due to the high proportion of pump users and parents believing that treatment with pumps implies less impact of the disease.

## Conclusions

The Norwegian version of the child and parent DISABKIDS instruments DCGM-37 and DDM-10 had acceptable reliability and validity in a population of children and adolescents with type 1 diabetes. DISABKIDS also discriminated between clinical characteristics that are important for disease management. We therefore suggest that HRQOL assessment with DISABKIDS may be of importance as a supplement to other clinical indicators in medical practice and research.

## Competing interests

The authors declare that they have no competing interests.

## Authors' contributions

DHF designed the study, collected and analyzed the data and drafted the manuscript. TM, TW-L, MG contributed to conceptualisation and design, data analysis, interpretation of results, drafting and revising the manuscript. KD-J and TS invited DHF to research collaboration with the Norwegian Childhood Diabetes Registry, contributed to conceptualisation and design of the study, collection of data and revising the manuscript. All authors read and approved the final manuscript.
